# Production and characterization of monoclonal antibodies for the detection of the hepatitis C core antigen

**DOI:** 10.3389/fmolb.2023.1225553

**Published:** 2023-07-13

**Authors:** Erick Joan Vidal-Alcántara, Vicente Mas, María Belén Yélamos, Julián Gómez, Rafael Amigot-Sánchez, Salvador Resino, Isidoro Martinez

**Affiliations:** ^1^ Unidad de Infección Viral e Inmunidad, Centro Nacional de Microbiología, Instituto de Salud Carlos III, Madrid, Spain; ^2^ Unidad de Biología Viral, Centro Nacional de Microbiología, Instituto de Salud Carlos III, Madrid, Spain; ^3^ Departamento de Bioquímica y Biología Molecular, Facultad de Ciencias Químicas, Universidad Complutense, Madrid, Spain; ^4^ Centro de Investigación Biomédica en Red en Enfermedades Infecciosas (CIBERINFEC), Instituto de Salud Carlos III, Madrid, Spain

**Keywords:** hepatitis C, core antigen, monoclonal antibody, rapid diagnostic test, screening

## Abstract

**Background:** Despite highly effective treatments to cure hepatitis C, almost 80% of chronically HCV-infected people are not treated, as they are unaware of their infection. Diagnostic rates and linkage to care must be substantially improved to reverse this situation. The HCV core antigen (HCVcAg) is a highly conserved protein that can be detected in the blood of HCV-infected patients and indicates active infection.

**Aim:** To produce murine monoclonal antibodies against HCVcAg suitable for rapid and inexpensive tests to detect HCV infection.

**Methods:** BALB/c mice were sequentially inoculated with purified recombinant HCVcAg from Gt1a, Gt3a, Gt4a, and Gt1b genotypes. Hybridomas producing the desired monoclonal antibodies were selected, and the reactivity of antibodies against HCVcAg from various genotypes was tested by Western blotting and dot blotting. The binding kinetics of the antibodies to purified HCVcAg was analyzed by surface plasmon resonance (SPR), and their ability to detect HCVcAg was tested by double antibody sandwich ELISA (DAS-ELISA).

**Results:** Four specific monoclonal antibodies (1C, 2C, 4C, and 8C) were obtained. 1C, 2C, and 4C recognized HCVcAg of all genotypes tested (Gt1a, Gt1b, Gt2a, Gt3a, and Gt4a), while 8C did not recognize the Gt2a and Gt3a genotypes. Based on SPR data, the antibody-HCVcAg complexes formed are stable, with 2C having the strongest binding properties. DAS-ELISA with different antibody combinations easily detected HCVcAg in culture supernatants from HCV-infected cells.

**Conclusion:** Specific and cross-reactive anti-HCVcAg monoclonal antibodies with strong binding properties were obtained that may be useful for detecting HCVcAg in HCV-infected samples.

## Introduction

Around 70% of people infected with the hepatitis C virus (HCV) develop chronic hepatitis C (CHC). The World Health Organization (WHO) estimates that 58 million people suffer from CHC worldwide and that 1.5 million new infections occur yearly (World Health Organization, 2022b). CHC progresses slowly over the years, during which liver inflammation and fibrosis develop, leading to cirrhosis in approximately 15%–30% of patients. When cirrhosis is established, the infection can progress to end-stage liver disease and hepatocellular carcinoma in 1%–3% of cases (Spearman et al., 2019). In 2019, around 290,000 people died due to complications resulting from HCV infection.

HCV is transmitted by direct contact with infected blood, so there are groups in which the proportion of infected people is higher, for example, those who share needles during intravenous drug use or those who have sexual practices with a risk of bleeding.

Although no vaccine against HCV exists, a highly effective treatment based on direct-acting antivirals (DAAs) can cure more than 95% of HCV-infected patients (Ponziani et al., 2017). Based on this, the WHO intends to eliminate hepatitis C as a public health problem by 2030. To do this, it has set the goal of diagnosing 90% of people infected with HCV and treating 80% of them (World Health Organization, 2021). However, since hepatitis C is mainly asymptomatic for several years after initial infection, almost 80% of infected people are unaware that they are infected. Consequently, all these people are not treated and can transmit the virus. In 2019, only 21% of the estimated 58 million people with chronic hepatitis C worldwide were diagnosed. Furthermore, between 2015 and 2019, only 62% (9.4 million) of the diagnosed people were treated. These data clearly show that the proportion of people diagnosed and treated must be substantially increased to achieve the WHO target. This requires large-scale screening efforts, especially in developing countries and populations at high risk of infection, such as people who inject drugs, men who have sex with men, incarcerated people, the homeless, etc. (World Health Organization, 2022a; World Health Organization, 2022b). Many of these people have limited access to health services, making the screening process difficult.

Currently, the standard diagnosis of hepatitis C is based on a first HCV antibody detection test which, if positive, requires confirmation of active infection through a second test to detect viral RNA. This methodology involves time, trained personnel, and complex laboratory equipment, as well as being expensive. Therefore, its performance is limited when applied at the population level and in risk groups that are difficult to access. Furthermore, during this procedure, many patients lose follow-up (Oru et al., 2021). Therefore, developing a rapid, inexpensive, and easy-to-use diagnostic test at patient care points (outpatient clinics, clinics, etc.) or for self-testing is necessary.

The HCV core antigen (HCVcAg) is a highly conserved protein between the different HCV genotypes (Bukh et al., 1994; Hitomi et al., 1995) and, like the RNA of the virus, is detected in the blood of patients with active infection (Moradpour and Penin, 2013; Laperche et al., 2015). Furthermore, HCVcAg levels correlate well with viral RNA (Galli et al., 2018). Therefore, the detection of HCVcAg is considered adequate for HCV screening and diagnosis. HCVcAg detection-based tests may be cheaper, faster, and easier to use than those that detect viral RNA, facilitating access to hepatitis C screening for many people, including at-risk populations (Aguilera et al., 2020). Additionally, HCV RNA degrades rapidly and can lead to false negatives (Wang et al., 2021).

Rapid tests that detect HCV RNA or HCVcAg in blood already exist or are in the approval process (Gay, 2020). However, none completely meets all the requirements (speed, low cost, easy use) that would be desirable for use at the point of care or self-testing.

Rapid diagnostic antigen tests (RDAT) are usually based on detecting the antigen by specific antibodies. In this study, we report the generation and characterization of four cross-reactive anti-HCVcAg monoclonal antibodies suitable for HCVcAg detection, as shown by double antibody sandwich ELISA (DAS-ELISA).

## Materials and methods

### Ethics statement

Mice were handled according to the regulations of Spanish and European legislation. Protocols were approved by the Ethics Committee of “Instituto de Salud Carlos III” (Ref.: CBA 10_2019-v3).

### Cells

Huh7.5 cells derived from human hepatoma were obtained from Apath LLC (Brooklyn, NY, United States), and Huh7.5.1 clone 2 was kindly provided by Dr. Francis V. Chisari (The Scripps Research Institute, La Jolla, CA, United States). Cell lines were maintained in Dulbecco’s Modified Eagle Medium (DMEM; Lonza, Basel, Switzerland), supplemented with 10% fetal bovine serum (FBS; Biological Industries, Beit Haemek, Israel), 4 mM L-glutamine (Lonza), and antibiotics (100 U/mL penicillin, 100 U/mL streptomycin; Lonza) at 37°C, 5% CO_2_.

### Chimeric HCV viruses and cell infections

The plasmid encoding the JFH1 genome (Gt2a) was obtained from Apath LLC (Wakita et al., 2005). The plasmids encoding JFH1-based chimeric viruses containing the Core-NS2 region of Gt1a (H77/JFH1) (Scheel et al., 2008), Gt1b (J4/JFH1) (Gottwein et al., 2009), Gt3a (S52/JFH1) (Gottwein et al., 2007), and Gt4a (ED43/JFH1) (Scheel et al., 2008) were kindly provided by Jens Bukh (Copenhagen University Hospital, Copenhagen, Denmark). Cell culture infectious HCVs (HCVcc) were produced from plasmid-transcribed RNAs and titrated as previously reported (Sepulveda-Crespo et al., 2022).

Huh7.5 clone 2 cells were infected at a multiplicity of infection (moi) of 0.01 focus-forming units per cell. The cells were then passed every 2–3 days until the virus titers reached maximum levels. At this point, cells were scraped and pelleted by low-speed centrifugation. Culture supernatants were stored at −80°C, and cell pellets were washed with PBS and resuspended in extraction buffer (10 mM Tris-HCl, pH 7.6, 5 mM EDTA, 140 mM NaCl, 1% Triton X-100, 1% sodium deoxycholate, and 0.1% SDS).

### Expression and purification of recombinant HCVcAg

**FIGURE 1:**
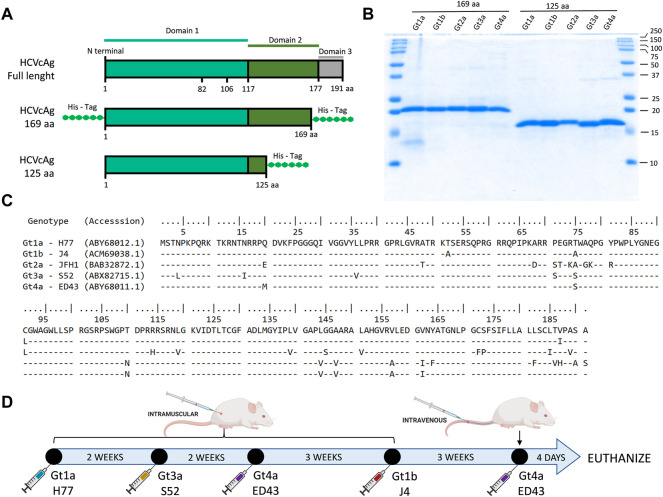
HCVcAg structure and primary sequences and immunization schedule. **(A)** Two different sets of truncated forms of HCVcAg (residues 1-125 and residues 1-169) were produced. **(B)** SDS-PAGE of purified HCVcAg (169aa and 125aa). **(C)** Alignment of the HCVcAg of different genotypes. **(D)** Mice immunization schedule. The mice were sequentially immunized with HCVcAg (169aa) of different genotypes.

Two sets of truncated forms of the HCVcAg (amino acid residues 1-125 and 1-169) were produced (Figures 1A–C). DNA sequences encoding the HCVcAg of five different genotypes, Gt1a (H77; GenBank accession no: EU363761), Gt1b (J4; accession no: FJ230881), Gt2a (JFH1; accession no: AB047639), Gt3a (S52; accession no: EU204645), and Gt4a (ED43; accession no: EU36376 0) with the addition of two six-histidine tags (His tag) at the 5′and 3′ ends (HCVcAg 1-169) or one His tag at the 3′ end (HCVcAg 1-125) were amplified by PCR and cloned in the *E. coli* expression vector pET21d (Novagen) to obtain the recombinant HCVcAg[1-125] and HCVcAg[1-169] plasmids. Two His tag additions to the 1-169 constructions were necessary to stabilize the expressed recombinant proteins against degradation.

*Escherichia coli* strain C41(DE3) cells were transformed with the corresponding recombinant plasmids. A single colony was selected and used to inoculate 150–800 mL of Luria-Bertani media supplemented with 100 μg/mL ampicillin. This culture was grown to an optical density at 600 nm of 0.6, and then IPTG (Sigma) was added to a final concentration of 0.5 mM and incubated at 37°C for 5 h to induce protein expression. Cells were harvested by centrifugation at 7,400 g for 10 min, and cell pellets were resuspended in denaturing buffer, 20 mM Tris, 50 mM NaCl, 6 M Guanidinium Chloride (GdmCl) pH 8.0, and lysed by tip sonication. The lysate was clarified by centrifugation at 27,000 g for 60 min.

Recombinant proteins from the supernatant were purified under denaturing conditions using a single affinity chromatography step in a 6BCL-QHNi High-Density Nickel column (ABT) equilibrated with denaturing buffer. Once the protein solution had entered the column, it was washed with a denaturing buffer containing 10 mM imidazole and later with the same buffer containing 30 mM imidazole. The proteins were eluted with denaturing buffer containing 200 mM imidazole. After elution, imidazole and GdmCl were readily removed from the protein solution using a PD-10 desalting column (GE Healthcare) equilibrated in 20 mM Tris, 50 mM NaCl, pH 8.0 buffer. The purified and renatured recombinant proteins were aliquoted and stored at −20°C. The presence of the HCVcAg was monitored throughout the purification by SDS-PAGE.

### Mouse immunization and hybridoma production and selection

BALB/c female mice between 6 and 8 weeks of age were inoculated intramuscularly with 10 μg of purified recombinant HCVcAg mixed with adjuvant (CpG Magic Mouse Adjuvant, Creative Diagnostics). Successive doses of HCVcAg from different genotypes were inoculated at 2–3 weeks intervals (Figure 1D). Four days after the last dose was administered, the mice were euthanized by isoflurane inhalation, and the spleen cells fused with Sp2-0 myeloma cells using polyethylene glycol (Stemcell Technologies). Hybridomas were seeded in 96 well plates and selected in Clonacell-HY selection medium (Stemcell Technologies) supplemented with HAT (hypoxanthine, aminopterin, and thymidine). After 7 days, the hybridomas were transferred to Clonacell-HY growth medium E (Stemcell Technologies). The presence of antibodies in hybridoma culture supernatants was analyzed by ELISA against purified HCV H77 HCVcAg and cell extracts from cells infected with H77 (Gt1a) recombinant HCV. Positive cultures were recloned at least twice by limiting dilution, expanded, and stored in liquid nitrogen.

### Antibody purification, concentration, labeling, and isotyping

The antibodies were purified from the hybridoma culture supernatant using CaptureSelect LC-kappa (mur) affinity resin (Catalog # 191315005, Thermo Scientific), concentrated (Clean-Up kit, ab102778, Abcam), and HRP-labeled (Lightning-link, ab102890, Abcam), following the manufacturer’s instructions.

The antibody isotype was determined using the Pro-Detect™ Rapid Antibody Isotyping Assay Kit, mouse (Catalog # A38550, Thermo Scientific).

### Western blotting and dot-blotting

Two hundred ng of purified recombinant proteins were separated in SDS-15% PAGE gels and transferred to an immobilon-P membrane (Millipore). The membrane was incubated for 1 hour with primary antibodies from selected hybridoma clones (1C, 2C, 4C, and 8C). Horseradish peroxidase-linked anti-mouse Ig (ab97040, Abcam) was used as a secondary antibody. Proteins were visualized by chemiluminescence using Pierce ECL Western Blot Substrate (Catalog # 32209, Thermo Fisher Scientific) on a ChemiDoc MP Imaging System instrument (Bio-Rad).

The amounts of proteins loaded on the gels were previously normalized in cell extracts according to their HCVcAg content using a pool of 1C, 2C, and 4C antibodies.

For dot blot assays, 3 µL of PBS-0.05% Tween20 containing 200 ng of purified recombinant proteins or normalized amounts of cell extracts were spotted on immobilon-P membranes (Millipore). The membranes were treated as described for Western blotting.

### Surface plasmon resonance (Biacore)

All the assays were run in a Biacore T200 instrument (Cytiva) using the recombinant HCVcAg as captured ligands and the antibodies as analytes. The Cytiva His-Capture kit (#28-9950-56) was used for covalently binding an anti-6xHis monoclonal antibody (11,000 response units) to the sample and the reference cells of a CM5 chip (Cytiva, #BR100530). Approximately 70 RUs of the recombinant HCVcAg proteins were captured in the sample cell by the anti-6xHis mAb. To reduce ligand heterogenicity, the captured HCVcAg proteins contained the first N-terminal 125 aa and just one histidine tag at the C-terminal end. In binding experiments, the antibodies were injected in a multicycle format using nine different antibody concentrations (0.39–100 nM). Calculating affinity or kinetic parameters was impossible since binding data was unsuccessfully fitted to any affinity or kinetic model. Therefore, the binding properties for each interaction were evaluated by early (20 s after the end of antibody injection) and late (280 s after the end of antibody injection) stability points achieved at the highest antibody concentration used (100 nM). For competition assays, the binding points (5 s before the end of antibody injection) achieved at the 12.5 nM antibody concentration were evaluated after a previous competitor injection. The injections of antibodies as competitors were performed at different concentrations (6.25–200 nM) and were prolonged for 20s without a dissociation step. All antibody injections were carried out using a flow rate of 50 μL/min. Regeneration between cycles was performed by 60s injections of 10 mM Glycine pH 1.5 solution at a 30 μL/min flow rate.

### ELISA and double antibody sandwich ELISA (DAS-ELISA)

For antibody screening, 96-well microtiter plates were coated for indirect ELISA with 20 ng per well of purified recombinant proteins (169 aa versions) or protein extracts from cells infected with JFH1 recombinant HCV. After blocking with PBS-TW20 0.05% supplemented with 5% porcine serum (26250-084, Thermo Fisher Scientific), primary antibodies (supernatants from hybridoma or purified antibodies 1C, 2C, 4C, and 8C) were added, and plates were incubated for 1 h at RT. Horseradish peroxidase-linked anti-mouse Ig (ab97040, Abcam) was used as the secondary antibody. Lastly, the substrate (OPD, ref. P8287, Sigma-Aldrich) was added, and the optical density was read at 493 nm. Extensive washing with water was done after each step.

For DAS-ELISA, 96-well microtiter plates were coated with 500 ng per well of purified capture antibodies. After blocking with PBS-5% BSA, antigens (40 ng of purified HCVcAg, concentrated supernatants from infected cells) were added, and plates were incubated for 30 min at RT. Subsequently, an HRP-conjugated detection antibody was added and incubated for 1 hour at RT, followed by substrate (OPD, ref. P8287, Sigma-Aldrich). Extensive washing with water was done after each step. The optical density was read at 493 nm.

Infected and non-infected control cell supernatants, obtained as described in the “Chimeric HCV viruses and cell infections” section, were concentrated 15 times using Vivaspin^®^ 20, 100,000 MWCO PES ultrafiltration concentrator units (VS 2041, SARTORIUS). Virus titers in concentrated supernatants were determined as focus-forming units per milliliter (FFU/mL) after staining with the anti-NS5A 9E10 antibody (a gift from Charles Rice; The Rockefeller University, New York, NY, United States), goat anti-mouse IgG-horseradish peroxidase (HRP) secondary antibody (Abcam, Cambridge, United Kingdom) and 3-amino-9-ethylcarbazole (AEC; Alfa Aesar, Heysham, United Kingdom) (Sepulveda-Crespo et al., 2022).

Before being tested, culture supernatants were solubilized by adding Triton X-100 (ref. T8532, Sigma-Aldrich) to a final concentration of 1%.

Commercial mouse anti-His tag (MCA1396) and anti-HCVcAg C7-50 (ab2740) antibodies were purchased from Bio-Rad and Abcam, respectively.

## Results

### Hybridoma selection

Five mice were immunized following the protocol outlined in Figure 1, and the hybridoma selection process is detailed in Supplementary Table S1. On average, each mouse yielded approximately 1300 hybridoma clones. These clones were screened using an ELISA assay, wherein they were simultaneously tested against purified recombinant HCVcAg H77 (Gt1a) and HBV Pre-S (utilized as a negative control). In some cases, complementary ELISA screening was performed by simultaneously testing the clones against protein cell extracts obtained from cells infected with the recombinant HCV (H77) and mock-infected cells. A selection of 10–28 hybridomas per mouse exhibiting high reactivity towards HCVcAg and no reactivity towards HBV Pre-S were chosen for further cloning.

Ultimately, a total of eight clones were selected for further analysis (Supplementary Table S1). Clones 5C, 6C, and 7C are not described in this manuscript, as they recognized the recombinant HCVcAg but not the HCVcAg from HCV-infected cells.

It is important to emphasize that despite initial screenings yielding a substantial number of positive clones, most of them lost their ability to produce antibodies during subsequent cloning rounds. This unexpected result may be due to genomic instability in hybridoma clones caused by their hyperploidy (Andreeff et al., 1985).

### Reactivity of antibodies with HCVcAg of distinct HCV genotypes

**FIGURE 2:**
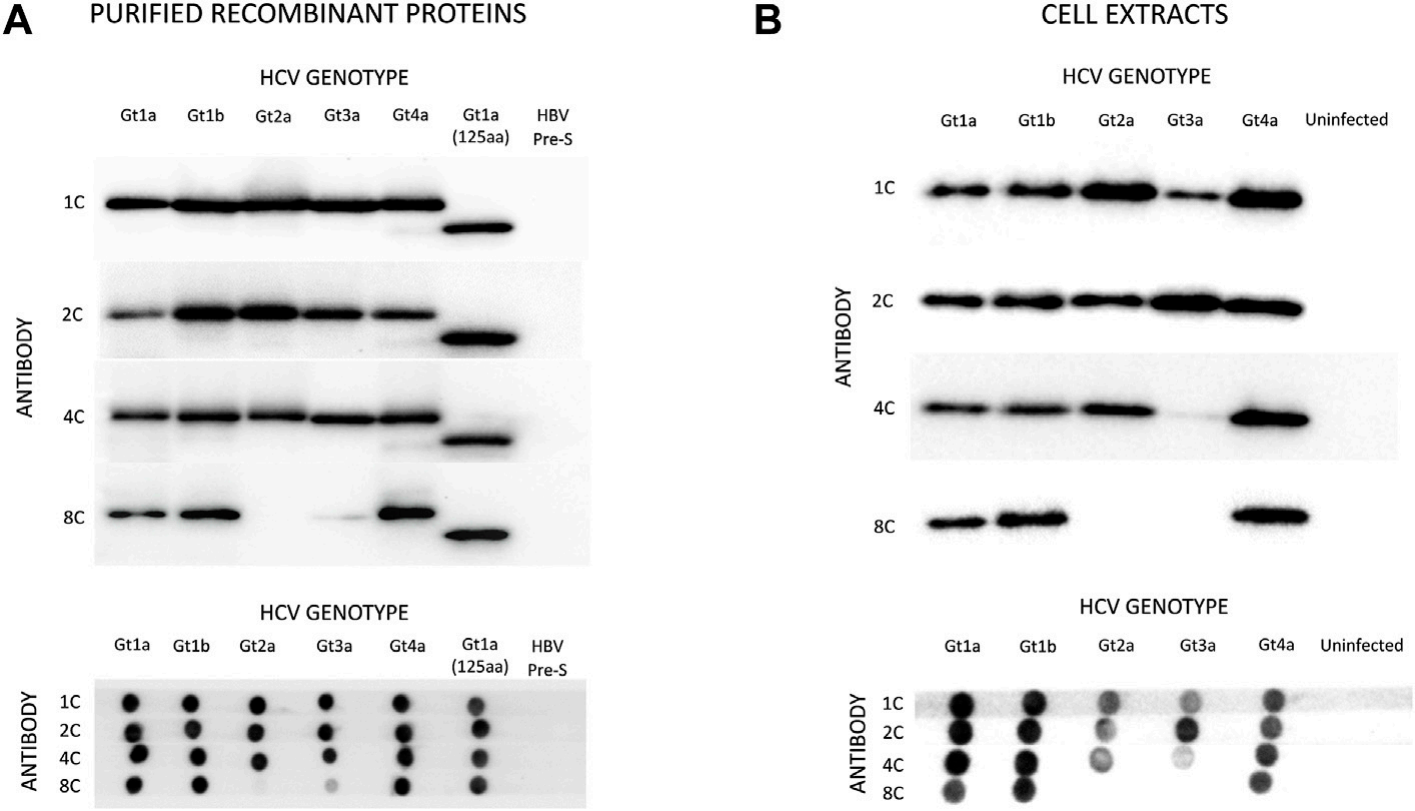
Reactivity of monoclonal antibodies (1C, 2C, 4C, and 8C) against HCVcAg of different genotypes (Gt1a, Gt1b, Gt2a, Gt3a, Gt4a). The antigen-antibody interaction was visualized by Western blotting (upper panel) or dot-blotting (lower panel). **(A)** Two hundred nanograms of purified recombinant HCVcAg were assayed in all cases. The recombinant HCVcAg comprises the first N-terminal 169 aa of the HCV core protein. A short version of Gt1a HCVcAg (first N-terminal 125 aa) was also included. **(B)** Reactivity of the monoclonal antibodies against HCVcAg extracted from HCV-infected cells. Huh7.5.1 cells were infected with five HCV recombinant viruses expressing different HCVcAg genotypes. The antigen-antibody interaction was visualized by Western blotting (upper panel) or dot-blotting (lower panel). The amounts of proteins loaded on the gels were previously normalized according to their HCVcAg content using a pool of 1C, 2C, and 4C antibodies. Abbreviations: HBV PreS, Hepatitis B virus PreS protein, a six-histidine tagged protein used as a negative control.

Four monoclonal antibodies were selected that specifically recognize HCVcAg of different genotypes: 1C (IgG2b), 2C (IgG2a), 4C (IgG2b), and 8C (IgG2b). Western blot (WB) and dot-blot (DB) assays showed that antibodies 1C, 2C, and 4C specifically reacted with purified HCVcAg from HCV genotypes Gt1a, Gt1b, Gt2a, Gt3a, and Gt4a (Figure 2A). However, antibody 8C did not react with HCVcAg of genotype Gt2a and barely recognized the protein of genotype Gt3a. The four monoclonal antibodies were directed against epitopes placed in the first 125 aa of HCVcAg since they reacted with a truncated version of the protein comprising only those amino acids (Figure 2A).

Almost identical results were obtained when antibodies were tested against protein extracts from Huh7.5.1 cells infected with chimeric HCV expressing HCVcAg of the same genotypes (Figure 2B). However, antibody 4C reacted poorly with HCVcAg of the Gt3a genotype in this case (Figure 2B). One possible explanation for the differences observed between purified proteins and proteins from cell extracts is the occurrence of post-translational modifications in the HCVcAg of Gt3a during infection. Such modification could impact the accessibility of 4C to the epitope. It is worth noting that these modifications are not anticipated to take place in *E. coli*, where recombinant proteins are expressed. Various post-translational modifications have been reported for HCVcAg in human cells, including phosphorylation, ubiquitination, and palmitoylation (Hundt et al., 2013).

### Test of antibody-HCVcAg interaction by surface plasmon resonance (SPR)

The binding of monoclonal antibodies to purified HCVcAg was measured by SPR (The raw sensorgrams are shown in Supplementary Figure S1). This technique confirmed that the 8C antibody did not bind Gt2a and poorly Gt3a HCVcAg (Supplementary Figure S1).

**FIGURE 3:**
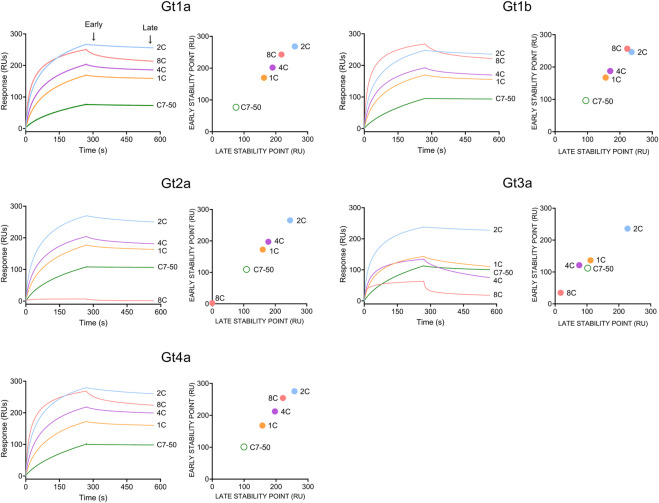
Binding of monoclonal antibodies (1C, 2C, 4C, 8C, and C7-50) to purified recombinant HCVcAg of different genotypes (Gt1a, Gt1b, Gt2a, Gt3a and Gt4a). The antibody-antigen interaction was measured by surface plasmon resonance. Sensorgrams corresponding to 100 nM antibody concentrations are shown. Early (20 s after the end of antibody injection) and late (20 s before the end of the dissociation period) stability points were calculated for each antibody-antigen interaction and plotted. Abbreviations: RU, response units; s, seconds.

Since binding data at the antibody concentration employed did not fit any affinity or kinetic binding model, probably due to the bivalency of antibodies and/or the presence of different oligomerization/conformational states in HCVcAg preparations, the binding properties for each antibody/protein interaction were determined by response values corresponding to early and late stability points achieved with the highest antibody concentration used (100 nM, Figure 3).

The strongest interaction was observed with the 2C antibody for all proteins, followed by 8C > 4C > 1C > C7-50 (Figure 3). As stated above, 8C did not interact with Gt2a and poorly with Gt3a HCVcAg. Additionally, the early and late stability points of the 1C and 4C antibodies with Gt3a HCVcAg were lower than those with the other proteins (Figure 3). It is important to remark that in all the cases where antibody/protein interaction was detected, the complexes formed were stable, which shows the suitability of these antibodies for developing DAS-ELISA or lateral flow immunochromatography tests.

### Antibody competition analysis

**FIGURE 4:**
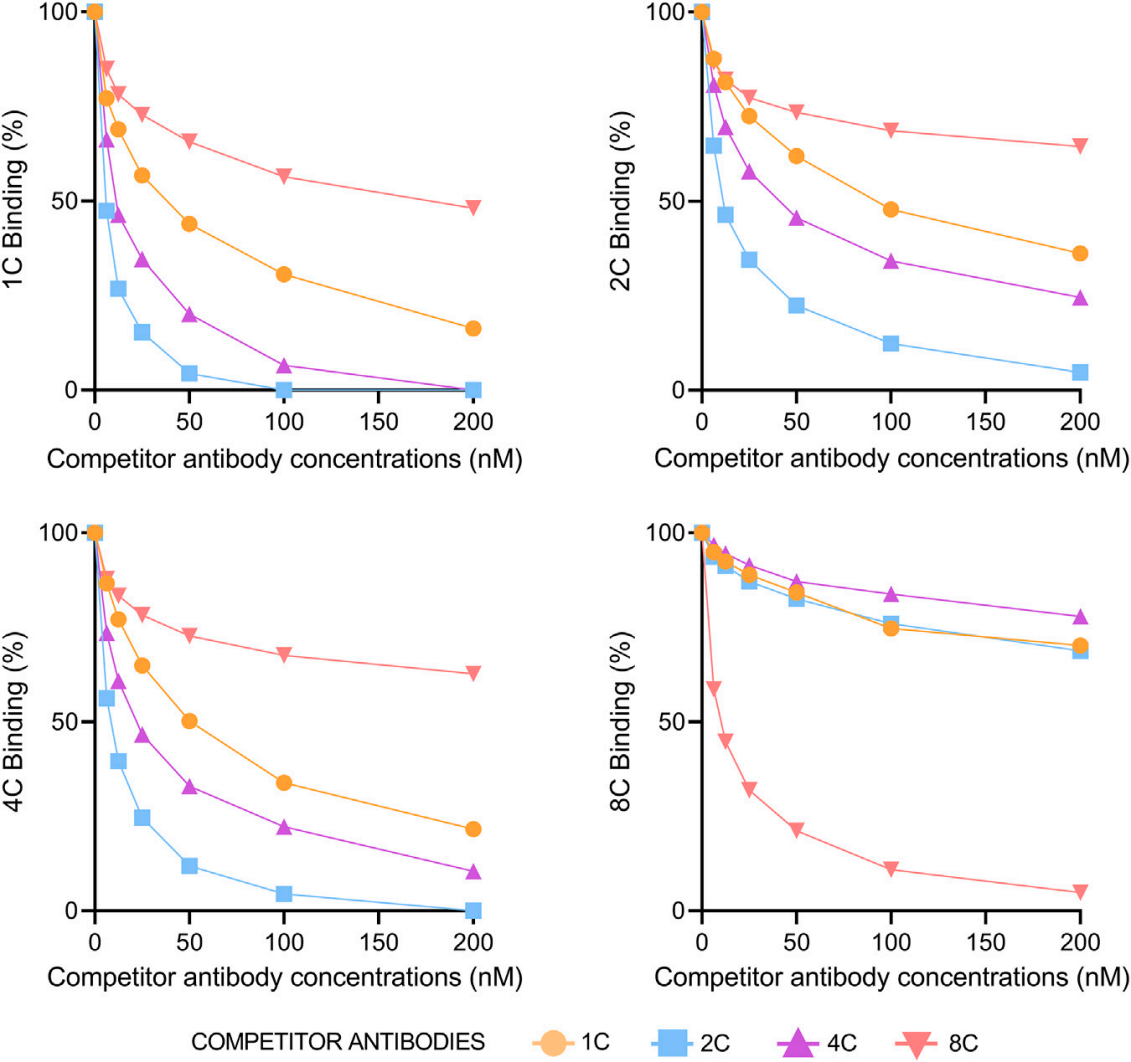
Competition of antibodies for binding to HCVcAg. The binding of each monoclonal antibody (1C, 2C, 4C, and 8C) at 12.5 nM concentration to HCVcAg (Gt1b, 125 aa) was assayed by surface plasmon resonance in the presence of increasing concentrations of the same or different competitor antibodies. The percentage of binding, determined by the binding points (5 s before the end of antibody injection), is represented relative to the condition in which the competitor antibody was absent. Abbreviations: nM, nanomolar.

The percentage of a particular antibody binding to HCVcAg Gt1b (125 aa) was measured at increasing concentrations of a competitor antibody. Figure 4 shows that antibodies 1C, 2C, and 4C competed with each other but not with 8C. The strongest competitor antibody was 2C, probably due to its stronger binding properties. Sensorgrams of the competition assays are shown in Supplementary Figure S2. We also performed a competition assay with the anti-HCVcAg commercial antibody C7-50, which competed with all other antibodies (1C, 2C, 4C, and 8C), indicating that it is directed against an epitope that overlaps with those recognized by them (Supplementary Figure S3).

### DAS-ELISA for HCVcAg detection

The ability of the antibodies to work in a DAS-ELISA was tested with two different samples: 1) purified recombinant proteins, 2) culture supernatants from *in vitro* HCV-infected cells.

**FIGURE 5:**
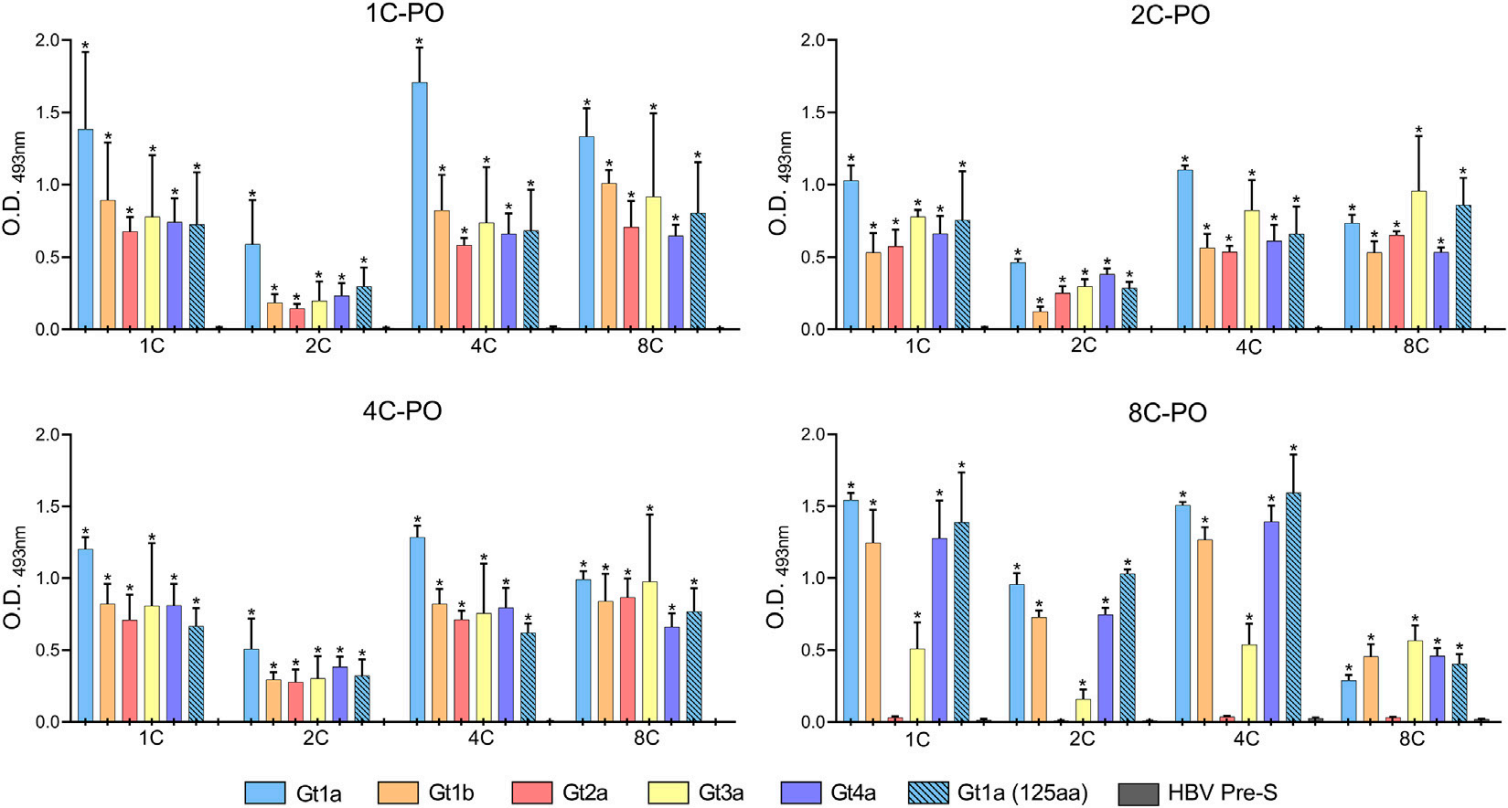
DAS-ELISA for the detection of purified recombinant HCVcAg (genotypes Gt1a, Gt1b, Gt2a, Gt3a, and Gt4a) with different combinations of capture antibodies (shown on the X-axis) and horseradish peroxidase-conjugated detection antibodies (1C-PO, 2C-PO, 4C-PO, and 8C-PO). The background (no capture antibody) was subtracted. Data represent the means and standard deviations of at least three independent experiments. Comparisons between conditions were made using a *t*-test. Statistically significant differences (*p* < 0.05) to HBV Pre-S were corrected by multiple testing using the false discovery rate (FDR) with the Benjamini and Hochberg method for each detection antibody, resulting in the calculation of a q-value. Statistically significant differences (q < 0.05) are denoted by an asterisk. Abbreviations: HBV, hepatitis B virus; O.D., optical density.

Most antibody combinations worked similarly with recombinant proteins (Figure 5). However, the 8C antibody did not detect HCVcAg Gt2a, and Gt3a was only slightly detected (Figure 5). Furthermore, when the best binder 2C antibody was used as the capture antibody, the signals were lower than with the other capture antibodies (Figure 5), probably due to its stronger binding properties that hinder the binding of detection antibodies. The DAS-ELISA detected HCVcAg at concentrations below 0.1 ng/μL (Supplementary Figure S4).

**FIGURE 6:**
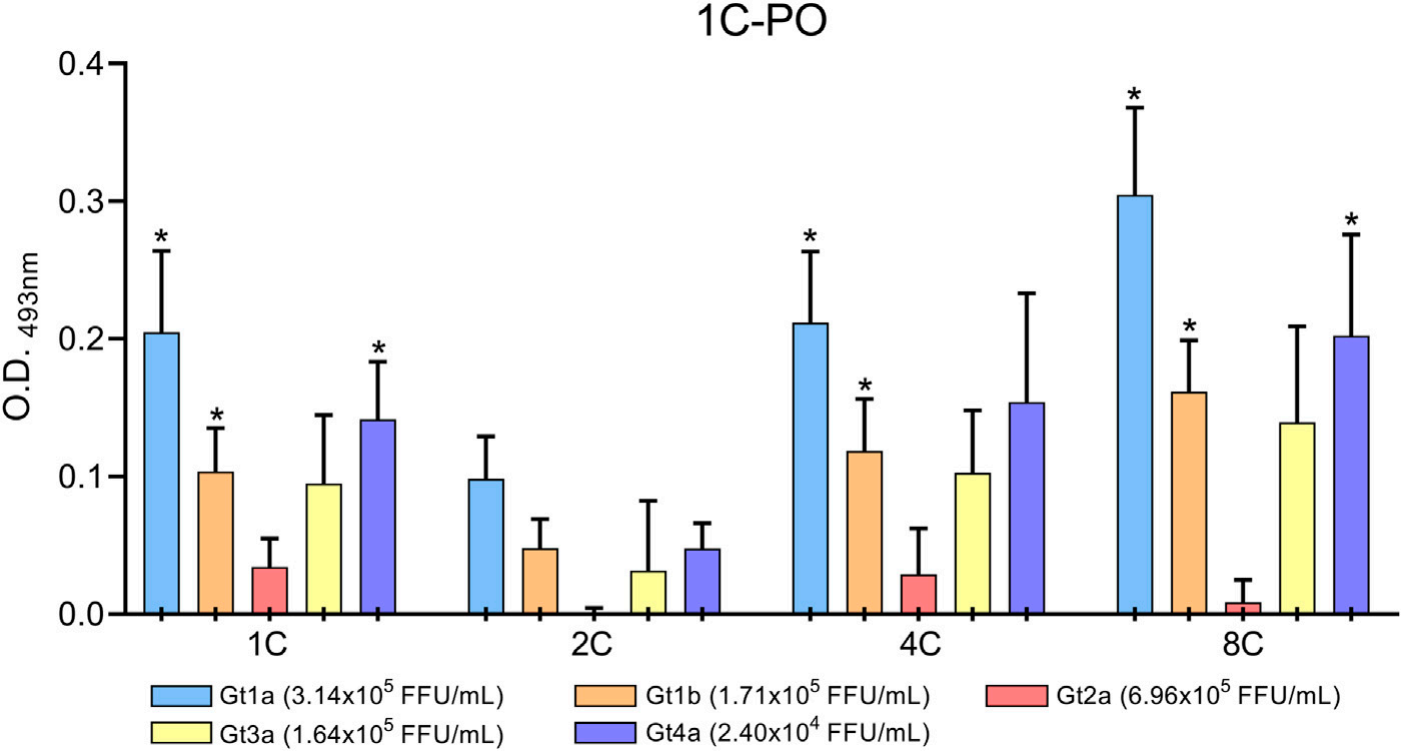
DAS-ELISA for HCVcAg detection (genotypes Gt1a, Gt1b, Gt2a, Gt3a, and Gt4a) from HCV-infected cell supernatants with combinations of different capture antibodies (shown on the X-axis) and horseradish peroxidase-conjugated detection antibody 1C (1C-PO). The background (no capture antibody) was subtracted. Data represent the means and standard deviations of at least three independent experiments. Comparisons between conditions were made using a *t*-test. Statistically significant differences (*p* < 0.05) to mock-infected cells were corrected by multiple testing using the false discovery rate (FDR) with the Benjamini and Hochberg method, resulting in the calculation of a q-value. Statistically significant differences (q < 0.05) are denoted by an asterisk. Abbreviations: FFU, focus forming units; O.D., optical density.

Culture supernatants of HCV-infected cells were concentrated and assayed in the DAS-ELISA to confirm that the monoclonal antibodies also detect HCVcAg proteins produced during an *in vitro* infection. HCVcAg Gt1a, Gt1b, Gt3a, and Gt4a were detected in the supernatants of infected cells when antibodies 1C, 4C, and 8C were used as capture antibodies (Figure 6). Despite the highest virus titer, the HCVcAg Gt2a signal was slightly above that of the supernatant of mock-infected cells only when 1C and 4C were used (Figure 6). However, these differences were not statistically significant. No significant differences were observed when the 2C antibody was used as a capture antibody (Figure 6).

## Discussion

This study describes four murine monoclonal antibodies that recognize HCVcAg from different HCV genotypes. They showed strong binding to the protein and easily detected it in DAS-ELISA assays.

Most people do not know they are infected with HCV and cannot be treated. Increasing diagnosis rates would reverse this situation. An HCVcAg-based diagnostic test would simplify, decentralize, and reduce HCV screening costs, thus promoting treatment access (Cresswell et al., 2015).

Although some HCVcAg-based tests are available, they do not meet all the requirements for widespread use at the point of care (Gay, 2020). The most common point-of-care tests rely on lateral flow immunochromatography (Clerc and Greub, 2010; Kozel and Burnham-Marusich, 2017; Nordgren et al., 2021). In this technique, antigen-antibody interaction plays a crucial role (Posthuma-Trumpie et al., 2009). Therefore, in order to increase the likelihood of antibodies being able to recognize native HCVcAg in samples from HCV-infected individuals, a careful selection of those antibodies capable of recognizing both HCVcAg from recombinant purified proteins and from extracts of infected cells was carried out. As a result of this process, antibodies 5C, 6C, and 7C were discarded.

The four antibodies described in this study may be ideal for the diagnosis based on HCVcAg since they have high specificity, strong binding properties, very stable interaction with HCVcAg, and cross-reactivity among different HCV genotypes. It is important to note that the binding association properties of these antibodies are, in most cases, stronger than that of the widely used commercial antibody C7-50 (Moradpour et al., 1996; Palmer et al., 2012). These remarkable characteristics of the antibodies are probably related to the immunization strategy, in which sequential inoculations with HCVcAg of different genotypes were carried out. In this way, the mice were successively primed for conserved epitopes (Torrents de la Pena et al., 2018). Additionally, the antibodies had enough time to undergo extensive affinity maturation (Doria-Rose and Joyce, 2015).

Although immunizations were carried out with recombinant HCVcAg comprising the first N-terminal 169 aa, all four antibodies are directed against the first 125 aa. This is not surprising since that domain contains mainly basic residues, while the region 126-169 is hydrophobic (Boulant et al., 2005). Furthermore, the basic domain has been reported to contain immunodominant epitopes, as seen in humans and mice (Sallberg et al., 1992a; Sallberg et al., 1992b; Ferroni et al., 1993; Goeser et al., 1994; Sallberg et al., 1994; Sato et al., 1994; Harase et al., 1995; Khanna and Ray, 1995; Park et al., 1995). Antibodies 1C, 2C, and 4C compete for HCVcAg binding, indicating that they are targeted to the same or close epitopes. However, these antibodies are not entirely equivalent since their reactivity with HCVcAg from different genotypes (mainly Gt3a) is different. On the contrary, the 8C antibody did not compete with the other antibodies, demonstrating that it recognizes a different epitope. This was confirmed by its particular reactivity pattern with HCVcAg from different genotypes. Finally, the commercial antibody C7-50 competed with all other antibodies (1C, 2C, 4C, and 8C), indicating that it is directed to an epitope that overlaps those recognized by them. The C7-50 epitope has been mapped between residues 21 to 40 of HCV-cAg (Moradpour et al., 1996). Interestingly, this protein segment overlaps different immunodominant regions (residues 19-26, 29-34, and 36–46) (Sallberg et al., 1994; Buratti et al., 1997) that may adopt different conformations and contain one or more epitopes (Buratti et al., 1997). Additionally, conformational heterogeneity may explain, at least in part, why the sensorgrams of the interaction between the antibodies 1C, 2C, 4C, and 8C with HCVcAg proteins did not fit any kinetics binding model in the SPR experiments.

Despite 1C, 2C, and 4C competing for HCVcAg binding, pair combinations of these antibodies work well in DAS-ELISA. This may be related to the fact that HCVcAg can form dimers (Supplementary Figure S5) (Lo and Ou, 1999; Boulant et al., 2005). In the SPR assays, where HCVcAg were used as captured ligands, increasing amounts of the competitor antibody block all available epitopes so that the binding antibody cannot attach to HCVcAg. This situation does not occur in DAS-ELISA assays, where a sandwich can be formed between the capture antibody, the dimeric HCVcAg, and the detection antibody.

The results of the DAS-ELISA developed in this work show that the antibodies might be suitable for rapid diagnostic antigen tests such as those based on lateral flow immunochromatography, a technique that also requires the formation of an antibody-antigen-antibody sandwich (Posthuma-Trumpie et al., 2009). These tests are widely used for other microorganisms, as they are fast and inexpensive and do not require complex equipment or trained personnel. It is important to note that although DAS-ELISA is not suitable for point-of-care tests, it may be helpful for laboratory screening. Our preliminary findings demonstrated that this test has the capability to detect HCVcAg in serum samples obtained from infected patients with an RNA viral load above 10^5^ IU/mL. It is worth emphasizing that the majority of untreated individuals with HCV infection commonly exhibit a high RNA viral load, typically ranging from 10^5^ to 10^7^ IU/mL (Zeuzem et al., 1996).

In conclusion, we have obtained four murine monoclonal antibodies that recognize HCVcAg with high specificity and affinity. Furthermore, antibodies are directed against epitopes conserved in the different HCV genotypes. Therefore, these antibodies are good candidates to include in rapid diagnostic tests for detecting HCVcAg.

## Figures and Tables

**Table:** 

**aa**	Amino acids
**BSA**	Bovine Serum Albumin
**CHC**	Chronic Hepatitis C
**DAAs**	Direct-Acting Antivirals
**DAS-ELISA**	Double-Antibody Sándwich Enzyme-Linked ImmunoSorbent Assay
**DB**	Dot-Blot
**DMEM**	Dulbecco’s Modified Eagle Medium
**EDTA**	Ethylenediaminetetraacetic Acid
**ELISA**	Enzyme-Linked ImmunoSorbent Assay
**FBS**	Fetal Bovine Serum
**FFU**	Focus-Forming Units
**GdmCl**	Guanidinium Chloride
**Gt**	Genotype
**HAT**	Hypoxanthine, Aminopterin, and Thymidine
**HBV**	Hepatitis B virus
**HCV**	Hepatitis C virus
**HCVcAg**	HCV core Antigen
**His-Tag**	Histidine Tag
**HRP**	Horseradish Peroxidase
**IPTG**	Isopropil ß-D-thiogalactopyranoside
**IU**	International Units
**moi**	Multiplicity of infection
**O.D**	Optical Density
**OPD**	o-Phenylenediamine Dihydrochloride
**PAGE**	Polyacrylamide Gel Electrophoresis
**PBS**	Phosphate-Buffered Saline
**PCR**	Polymerase Chain Reaction
**RDAT**	Rapid Diagnostic Antigen Tests
**RNA**	Ribonucleic Acid
**RU**	Response Units
**SDS**	Sodium Dodecyl Sulfate
**SPR**	Surface Plasmon Resonance
**Tris**	Tris(hydroxymethyl)aminomethane
**WB**	Western Blot
**WHO**	World Health Organization

## Data Availability

The original contributions presented in the study are included in the article/Supplementary Material, further inquiries can be directed to the corresponding author.

## References

[B1] Aguilera A. Alados J. C. Alonso R. Eiros J. M. Garcia F. (2020). Current position of viral load versus hepatitis C core antigen testing. Enferm. Infecc. Microbiol. Clin. Engl. Ed. 38 (Suppl. 1), 12–18. 10.1016/j.eimc.2020.02.003 32111360

[B2] Andreeff M. Bartal A. Feit C. Hirshaut Y. (1985). Clonal stability and heterogeneity of hybridomas: Analysis by multiparameter flow cytometry. Hybridoma 4 (3), 277–287. 10.1089/hyb.1985.4.277 2412947

[B3] Boulant S. Vanbelle C. Ebel C. Penin F. Lavergne J. P. (2005). Hepatitis C virus core protein is a dimeric alpha-helical protein exhibiting membrane protein features. J. Virol. 79 (17), 11353–11365. 10.1128/JVI.79.17.11353-11365.2005 16103187 PMC1193582

[B4] Bukh J. Purcell R. H. Miller R. H. (1994). Sequence analysis of the core gene of 14 hepatitis C virus genotypes. Proc. Natl. Acad. Sci. U. S. A. 91 (17), 8239–8243. 10.1073/pnas.91.17.8239 8058787 PMC44581

[B5] Buratti E. Di Michele M. Song P. Monti-Bragadin C. Scodeller E. A. Baralle F. E. (1997). Improved reactivity of hepatitis C virus core protein epitopes in a conformational antigen-presenting system. Clin. Diagn Lab. Immunol. 4 (2), 117–121. 10.1128/cdli.4.2.117-121.1997 9067642 PMC170488

[B6] Clerc O. Greub G. (2010). Routine use of point-of-care tests: Usefulness and application in clinical microbiology. Clin. Microbiol. Infect. 16 (8), 1054–1061. 10.1111/j.1469-0691.2010.03281.x 20670287

[B7] Cresswell F. V. Fisher M. Hughes D. J. Shaw S. G. Homer G. Hassan-Ibrahim M. O. (2015). Hepatitis C core antigen testing: A reliable, quick, and potentially cost-effective alternative to hepatitis C polymerase chain reaction in diagnosing acute hepatitis C virus infection. Clin. Infect. Dis. 60 (2), 263–266. 10.1093/cid/ciu782 25301216

[B8] Doria-Rose N. A. Joyce M. G. (2015). Strategies to guide the antibody affinity maturation process. Curr. Opin. Virol. 11, 137–147. 10.1016/j.coviro.2015.04.002 25913818 PMC4456294

[B9] Ferroni P. Mascolo G. Zaninetti M. Colzani D. Pregliasco F. Pirisi M. (1993). Identification of four epitopes in hepatitis C virus core protein. J. Clin. Microbiol. 31 (6), 1586–1591. 10.1128/jcm.31.6.1586-1591.1993 7686184 PMC265582

[B10] Galli C. Julicher P. Plebani M. (2018). HCV core antigen comes of age: A new opportunity for the diagnosis of hepatitis C virus infection. Clin. Chem. Lab. Med. 56 (6), 880–888. 10.1515/cclm-2017-0754 29702484

[B11] Gay B. (2020). Pipeline report. HCV diagnostics. New York: Treatment Action Group.

[B12] Goeser T. Muller H. M. Ye J. Pfaff E. Theilmann L. (1994). Characterization of antigenic determinants in the core antigen of the hepatitis C virus. Virology 205 (2), 462–469. 10.1006/viro.1994.1666 7526540

[B13] Gottwein J. M. Scheel T. K. Hoegh A. M. Lademann J. B. Eugen-Olsen J. Lisby G. (2007). Robust hepatitis C genotype 3a cell culture releasing adapted intergenotypic 3a/2a (S52/JFH1) viruses. Gastroenterology 133 (5), 1614–1626. 10.1053/j.gastro.2007.08.005 17983807

[B14] Gottwein J. M. Scheel T. K. Jensen T. B. Lademann J. B. Prentoe J. C. Knudsen M. L. (2009). Development and characterization of hepatitis C virus genotype 1-7 cell culture systems: Role of CD81 and scavenger receptor class B type I and effect of antiviral drugs. Hepatology 49 (2), 364–377. 10.1002/hep.22673 19148942

[B15] Harase I. Moriyama T. Kaneko T. Kita H. Nomura M. Suzuki G. (1995). Immune response to hepatitis C virus core protein in mice. Immunol. Cell Biol. 73 (4), 346–352. 10.1038/icb.1995.53 7493772

[B16] Hitomi Y. Mcdonnell W. M. Killeen A. A. Askari F. K. (1995). Sequence analysis of the hepatitis C virus (HCV) core gene suggests the core protein as an appropriate target for HCV vaccine strategies. J. Viral Hepat. 2 (5), 235–241. 10.1111/j.1365-2893.1995.tb00035.x 8745315

[B17] Hundt J. Li Z. Liu Q. (2013). Post-translational modifications of hepatitis C viral proteins and their biological significance. World J. Gastroenterol. 19 (47), 8929–8939. 10.3748/wjg.v19.i47.8929 24379618 PMC3870546

[B18] Khanna A. Ray R. (1995). Hepatitis C virus core protein: Synthesis, affinity purification and immunoreactivity with infected human sera. Gene 153 (2), 185–189. 10.1016/0378-1119(94)00782-n 7533115

[B19] Kozel T. R. Burnham-Marusich A. R. (2017). Point-of-Care testing for infectious diseases: Past, present, and future. J. Clin. Microbiol. 55 (8), 2313–2320. 10.1128/JCM.00476-17 28539345 PMC5527409

[B20] Laperche S. Nubling C. M. Stramer S. L. Brojer E. Grabarczyk P. Yoshizawa H. (2015). Sensitivity of hepatitis C virus core antigen and antibody combination assays in a global panel of window period samples. Transfusion 55 (10), 2489–2498. 10.1111/trf.13179 26013970 PMC4744653

[B21] Lo S. Y. Ou J. H. (1999). Expression and dimerization of hepatitis C virus core protein in *E. coli* . Methods Mol. Med. 19, 325–330. 10.1385/0-89603-521-2:325 21374373

[B22] Moradpour D. Penin F. (2013). Hepatitis C virus proteins: From structure to function. Curr. Top. Microbiol. Immunol. 369, 113–142. 10.1007/978-3-642-27340-7_5 23463199

[B23] Moradpour D. Wakita T. Tokushige K. Carlson R. I. Krawczynski K. Wands J. R. (1996). Characterization of three novel monoclonal antibodies against hepatitis C virus core protein. J. Med. Virol. 48 (3), 234–241. 10.1002/(SICI)1096-9071(199603)48:3<234::AID-JMV4>3.0.CO;2-9 8801283

[B24] Nordgren J. Sharma S. Olsson H. Jamtberg M. Falkeborn T. Svensson L. (2021). SARS-CoV-2 rapid antigen test: High sensitivity to detect infectious virus. J. Clin. Virol. 140, 104846. 10.1016/j.jcv.2021.104846 33971580 PMC8105081

[B25] Oru E. Trickey A. Shirali R. Kanters S. Easterbrook P. (2021). Decentralisation, integration, and task-shifting in hepatitis C virus infection testing and treatment: A global systematic review and meta-analysis. Lancet Glob. Health 9 (4), e431–e445. 10.1016/S2214-109X(20)30505-2 33639097 PMC7966682

[B26] Palmer B. A. Menton J. Levis J. Kenny-Walsh E. Crosbie O. Fanning L. J. (2012). The pan-genotype specificity of the hepatitis C virus anti-core monoclonal antibody C7-50 is contingent on the quasispecies profile of a population. Arch. Virol. 157 (11), 2235–2239. 10.1007/s00705-012-1418-4 22828781

[B27] Park H. J. Byun S. M. Ha Y. J. Ahn J. S. Moon H. M. (1995). Identification of immunodominant epitopes in the core and non-structural region of hepatitis C virus by enzyme immunoassay using synthetic peptides. J. Immunoass. 16 (2), 167–181. 10.1080/15321819508013556 7543117

[B28] Ponziani F. R. Mangiola F. Binda C. Zocco M. A. Siciliano M. Grieco A. (2017). Future of liver disease in the era of direct acting antivirals for the treatment of hepatitis C. World J. Hepatol. 9 (7), 352–367. 10.4254/wjh.v9.i7.352 28321272 PMC5340991

[B29] Posthuma-Trumpie G. A. Korf J. Van Amerongen A. (2009). Lateral flow (immuno)assay: Its strengths, weaknesses, opportunities and threats. A literature survey. Anal. Bioanal. Chem. 393 (2), 569–582. 10.1007/s00216-008-2287-2 18696055

[B30] Sallberg M. Pumpen P. Zhang Z. X. Lundholm P. Gusars I. Ruden U. (1994). Locations of antibody binding sites within conserved regions of the hepatitis C virus core protein. J. Med. Virol. 43 (1), 62–68. 10.1002/jmv.1890430112 7521899

[B31] Sallberg M. Ruden U. Wahren B. Magnius L. O. (1992a). Immune response to a single peptide containing an immunodominant region of hepatitis C virus core protein: The isotypes and the recognition site. Immunol. Lett. 33 (1), 27–33. 10.1016/0165-2478(92)90089-7 1385318

[B32] Sallberg M. Ruden U. Wahren B. Magnius L. O. (1992b). Immunodominant regions within the hepatitis C virus core and putative matrix proteins. J. Clin. Microbiol. 30 (8), 1989–1994. 10.1128/jcm.30.8.1989-1994.1992 1380007 PMC265429

[B33] Sato A. Sho Y. Nakamura H. Kunitomo T. Arima T. (1994). Immune responses of blood donors to peptides of various lengths and those with genotypic sequence variations corresponding to the N-terminal portion of the core protein of hepatitis C virus. J. Med. Virol. 44 (1), 88–91. 10.1002/jmv.1890440116 7528261

[B34] Scheel T. K. Gottwein J. M. Jensen T. B. Prentoe J. C. Hoegh A. M. Alter H. J. (2008). Development of JFH1-based cell culture systems for hepatitis C virus genotype 4a and evidence for cross-genotype neutralization. Proc. Natl. Acad. Sci. U. S. A. 105 (3), 997–1002. 10.1073/pnas.0711044105 18195353 PMC2242719

[B35] Sepulveda-Crespo D. Yelamos M. B. Diez C. Gomez J. Hontanon V. Torresano-Felipe F. (2022). Negative impact of HIV infection on broad-spectrum anti-HCV neutralizing antibody titers in HCV-infected patients with advanced HCV-related cirrhosis. Biomed. Pharmacother. 150, 113024. 10.1016/j.biopha.2022.113024 35483197

[B36] Spearman C. W. Dusheiko G. M. Hellard M. Sonderup M. (2019). Hepatitis C. Lancet 394 (10207), 1451–1466. 10.1016/S0140-6736(19)32320-7 31631857

[B37] Torrents De La Pena A. De Taeye S. W. Sliepen K. Labranche C. C. Burger J. A. Schermer E. E. (2018). Immunogenicity in rabbits of HIV-1 SOSIP trimers from clades A, B, and C, given individually, sequentially, or in combination. J. Virol. 92, e01957. 10.1128/JVI.01957-17 29367243 PMC5874403

[B38] Wakita T. Pietschmann T. Kato T. Date T. Miyamoto M. Zhao Z. (2005). Production of infectious hepatitis C virus in tissue culture from a cloned viral genome. Nat. Med. 11 (7), 791–796. 10.1038/nm1268 15951748 PMC2918402

[B39] Wang Y. Jie W. Ling J. Yuanshuai H. (2021). HCV core antigen plays an important role in the fight against HCV as an alternative to HCV-RNA detection. J. Clin. Lab. Anal. 35 (6), e23755. 10.1002/jcla.23755 33788295 PMC8183919

[B40] World Health Organization (2022a). Global health sector strategies on viral hepatitis 2016-2021. Avalaible at: http://apps.who.int/gb/ebwha/pdf_files/WHA69/A69_32-en.pdf?ua=1.

[B41] World Health Organization (2022b). Hepatitis C. Avalaible at https://www.who.int/news-room/fact-sheets/detail/hepatitis-c.

[B42] World Health Organization (2021). The global health sector strategies on, respectively, HIV, viral hepatitis and sexually transmitted infections 2022-2030. Geneva: World Health Organization. Licence: CC BY-NC-SA 3.0 IGO.

[B43] Zeuzem S. Franke A. Lee J. H. Herrmann G. Ruster B. Roth W. K. (1996). Phylogenetic analysis of hepatitis C virus isolates and their correlation to viremia, liver function tests, and histology. Hepatology 24 (5), 1003–1009. 10.1002/hep.510240505 8903367

