# Supplementary material for: Production and characterization of monoclonal antibodies for the detection of the hepatitis C core antigen
Source: Front Mol Biosci. 2023 Jul 13;10:1225553. doi: 10.3389/fmolb.2023.1225553 (PMC10374198; doi:10.3389/fmolb.2023.1225553)
Supplement: Supplementary file 1 [file Table1.docx]

Table I. Description of the hybridoma selection process

| Mouse | Nº of splenocytes | Nº of clones | Nº of selected Hybridomas | Screening | Antibody |
| --- | --- | --- | --- | --- | --- |
| 1 | 2,2x10^8^ | 992 | 25 | Purified HCVcAg (H77) / HBV Pre-S  HCV (H77) infected cells / mock-infected cells | 1C, 2C and 4C |
| 2 | 2,8x10^8^ | 1344 | 16 | Purified HCVcAg (H77) / HBV Pre-S  HCV (H77) infected cells / mock-infected cells |  |
| 3 | 2.3x10^8^ | 1344 | 10 | Purified HCVcAg (H77) / HBV Pre-S | 5C |
| 4 | 2.0x10^8^ | 960 | 11 | Purified HCVcAg (H77) / HBV Pre-S | 6C and 7C |
| 5 | 4.0x10^8^ | 1920 | 28 | Purified HCVcAg (H77) / HBV Pre-S | 8C |
