# Supplementary figures and images for: Production and characterization of monoclonal antibodies for the detection of the hepatitis C core antigen
Source: Front Mol Biosci. 2023 Jul 13;10:1225553. doi: 10.3389/fmolb.2023.1225553 (PMC10374198; doi:10.3389/fmolb.2023.1225553)

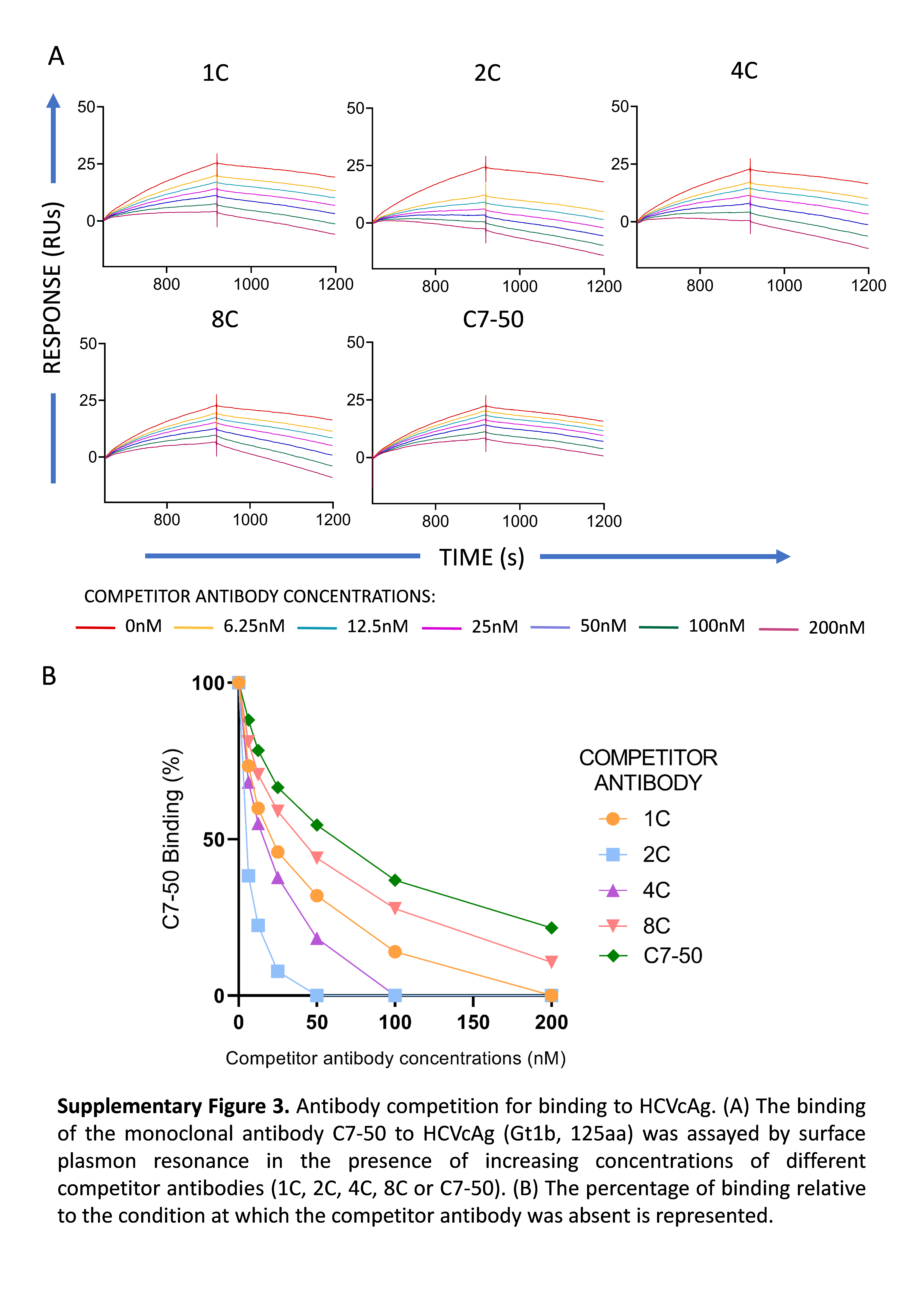

Supplement: Supplementary file 2 [file Image3.TIF]

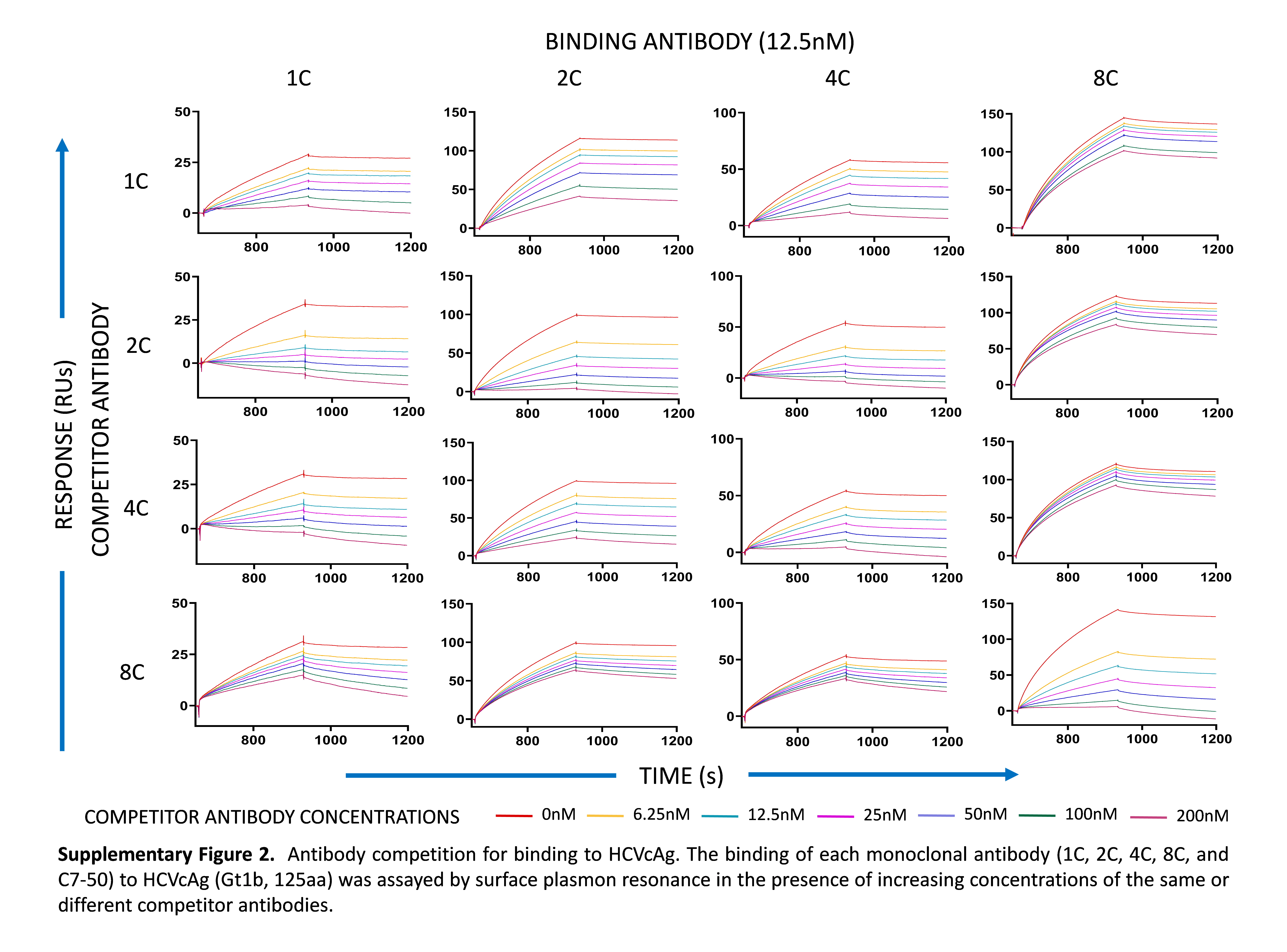

Supplement: Supplementary file 4 [file Image2.TIF]

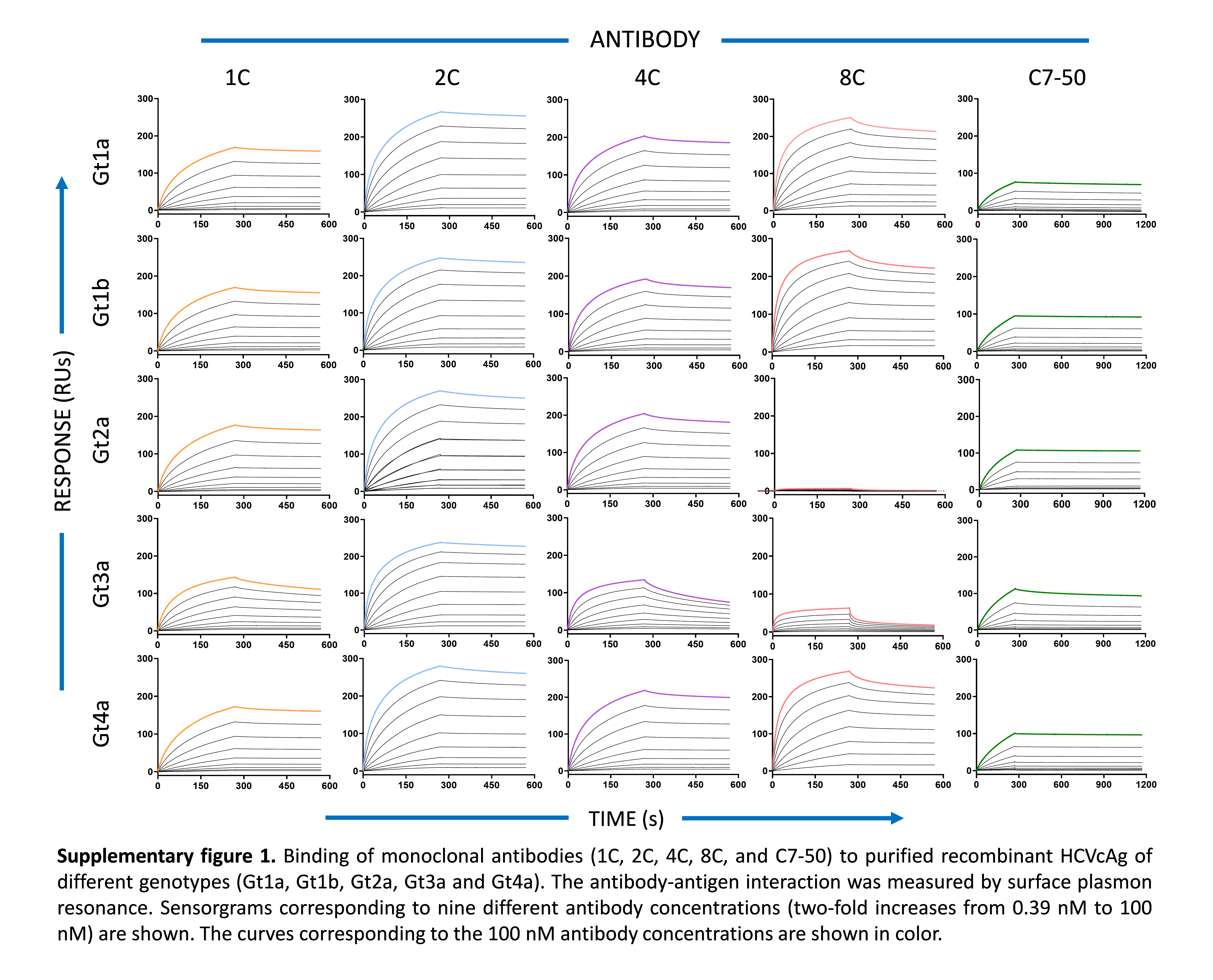

Supplement: Supplementary file 5 [file Image1.TIF]

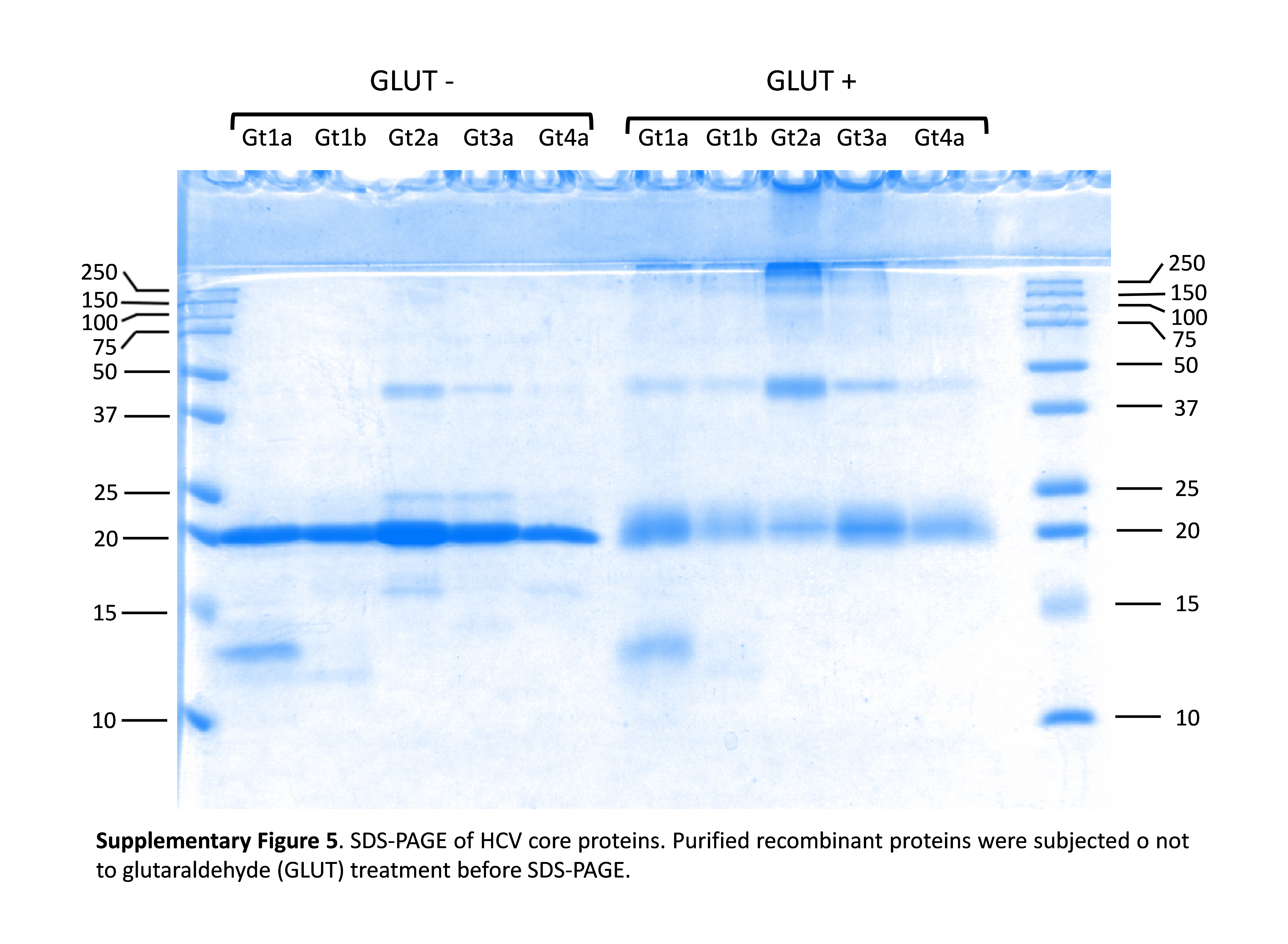

Supplement: Supplementary file 6 [file Image5.TIF]
